# Self-organized Sr leads to solid state twinning in nano-scaled eutectic Si phase

**DOI:** 10.1038/srep31635

**Published:** 2016-08-16

**Authors:** M. Albu, A. Pal, C. Gspan, R. C. Picu, F. Hofer, G. Kothleitner

**Affiliations:** 1 Graz Centre of Electron Microscopy, Steyrergasse 17/III, 8010 Graz, Austria; 2 Department of Mechanical, Aerospace and Nuclear Engineering, Rensselaer Polytechnic Institute, Troy, NY 12180, USA; 3 Institute for Electron Microscopy and Nanoanalysis, Graz University of Technology, Steyrergasse 17/III, 8010 Graz, Austria

## Abstract

A new mechanism for twin nucleation in the eutectic Al-Si alloy with trace Sr impurities is proposed. Observations made by sub-angstrom resolution scanning transmission electron microscopy and X-ray probing proved the presence of <110> Sr columns located preferentially at twin boundaries. Density functional theory simulations indicate that Sr atoms bind in the Si lattice only along the <110> direction, with preferential positions at first and second nearest neighbors for interstitial and substitutional Sr, respectively. Density functional theory total energy calculations confirm that twin nucleation at Sr columns is energetically favorable. Hence, twins may nucleate in Si precipitates after solidification, which provides a different perspective to the currently accepted mechanism which suggests twin formation during precipitate growth.

The past decades have witnessed a noticeable increase in research and improvement of light Al-Si cast alloys for use in domestic, military, automotive, and aerospace components^1^,^2^. Given the widespread (80%) use of this alloy system optimization of the microstructure is of crucial matter to ensure superior mechanical properties for the as-cast finished products^3^. Of particular importance are the nucleation and growth mechanisms of the eutectic Si phase which can be manipulated through changes in impurity concentration or kinetics of the eutectic reaction^4^,^5^,^6^,^7^,^8^,^9^,^10^,^11^. Several theories have been proposed to explain the mechanisms leading to refinement and twinning with the most broadly accepted ones being “impurity-induced twinning” (IIT)^6^ and the “twin plane re-entrant growth mechanism” (TPRE)^7^,^8^,^10^. Various experimental studies and sophisticated microscopic and tomographic investigations have been carried out for verification^12^,^13^,^14^,^15^,^16^,^17^. Recently, the twin formation mechanism which entails solute adsorption (along the <112>Si) and entrapment during Si growth was presented by Li^14^,^18^.

It has been concluded that none of the existing theories explains microstructural refinement and twinning induced by all atomic modifiers (Ba, Ca, Na, Sc or rare earths such as Yb, Y, Sb)^6^,^19^,^20^,^21^,^22^,^23^,^24^,^25^. Furthermore, twin formation in eutectic silicon and in presence of traces of impurities (<0.1 wt%) has also been observed upon alloy annealing^26^, which indicates that solute re-organization during the heat treatment may also lead to twin formation.

Density functional theory (DFT) has been used to study the stability of several known Al-Si-Sr phases^27^,^28^, of which some were known and some others have been newly identified as stable structures. Thermodynamic properties and vibration spectra of these intermetallics are also reported. Yue *et al*.^29^ used *ab-initio* molecular dynamics to study the structure of liquid Al-Si-Sr at the eutectic concentration of Al and Si. They indicate that the presence of Sr reduces the diffusion coefficient of both Al and Si in the melt and observed that Sr binds preferentially with Si.

This work targets the twinning mechanism in the presence of Sr using a combination of sub-angstrom resolution analytical scanning transmission electron microscopy (HR STEM) and *ab-initio* DFT calculations. Analytical evidence for the presence of Sr atomic columns, arranged only along certain crystallographic directions and at twin boundaries, is backed up by DFT total energy calculations, aiming to answer also whether a binding with the twin boundaries exists, and to shed some light on the energetic driving force for twin nucleation from such sites.

## Experimental Results

A high purity Al-Si alloy with 5 wt.% Si and 0.02 wt.% Sr containing eutectic Si particles were tilted in the {111}_Si_<110>_Si_//{111}_α-Al_<110>_α-Al_ direction and investigated by HR STEM. Care was taken to choose dose conditions under which beam induced Sr diffusion was negligible (sample preparation and microscope settings contained in Methods and Supplementary Material).

**Figure 1:**
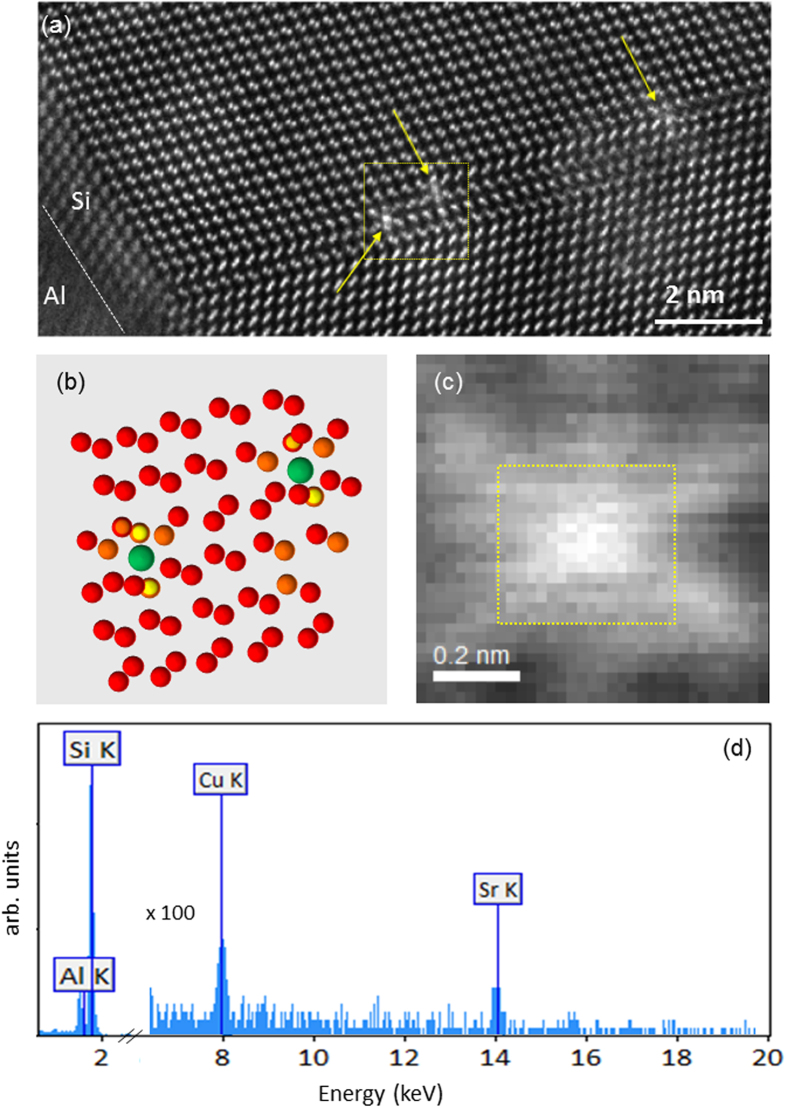
(**a**) HAADF STEM high resolution image (300 kV) of an eutectic Si particle tilted in the [110] zone axis. Yellow arrows indicate first order twins in {111} <112> direction associated with Sr columns. (**b**) Shows a qualitative representation of the Si and Sr columns intensities within the yellow square in Fig. 1(a) (Si - full contrast in red, Si - lower contrast in orange, Si - lowermost contrast in yellow-orange, and the Sr columns in green). (**c**) HAADF STEM image of the X-ray Spectrum Image (60 kV) from a “star-like” region in Fig. 1(a); (**d**) extracted spectrum from EDX –SI; the Sr atom column is marked with an yellow dashed square in Fig. 1(c). Strontium signal is about 100 times smaller than the silicon signal. The copper signal is from the TEM holder and the faint aluminum signal is more likely due to the fact that the signal is averaged through the thickness of the sample, and some Al matrix remained in the depth of the investigated area.

Viewed along the [110] zone axis (Fig. 1(a)), the silicon dumbbells of a Si particle (740 Å thick) and first order twins, growing in the {111} <112> direction, can be observed. Some twins extend to the interface with the aluminum matrix, while others initiate from brighter interstitial Sr atomic columns indicated by arrows in Fig. 1(a). The first neighboring Si dumbbell of the twin is rotated by 70.3° relative to the perfect Si lattice, resulting in a “star-like” appearance. Analytical imaging of the Sr columns by EDX, however, required different conditions due to Sr diffusion on the surface^30^, triggered by electron beam irradiation. To avoid Sr loss in the region investigated by X-ray Spectrum Imaging, low dose and cumulative acquisition conditions at 60 kV were chosen.

Chemical X-ray analysis presented in Fig. 1(c,d) confirmed the interstitial columns to be constituted of Sr atoms (detailed information about EDX acquisition is contained in STEM Methods and further spectra and images in Supplementary Material). Sr signal in Fig. 1(d) is about 100 times smaller than the Si signal. The Cu signal is from the TEM holder and the faint Al peak is more likely due to the fact that the signal is averaged through the thickness of the sample, and some Al matrix can be detected in the depth of the investigated area. Note that an absolute quantitative analysis of the Sr signal based on the EDX-SI, although in some cases possible, is here not reasonable given the low number of strontium atoms in one column. Around the strontium cores an intensity decrease can be observed, suggesting possible vacancies along [110] and leading to a possible model structure in Fig. 1(b). It should be mentioned however, that Sr could hardly ever be detected along the twins in the 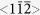 direction in the notably substitutional zigzag arrangement, as expected from the poisoning of the TPRE mechanism. Furthermore no significant HAADF contrast in the STEM image (contrast scales with the atomic number ~Z^1.7^) above background was found in the bulk, un-twinned Si. Moreover, since Al and Si have closed atomic numbers, the HAADF contrast cannot be used to recognize the diffuse distributed Al atoms. Qualitative HAADF image simulations (QSTEM software) reveal that a full replacement of Si by Al still yields a contrast reduction of only 30% (supporting Figure S2). This means that for any level of Si replacement by Al, intensity is visible and column information does not vanish.

Previous STEM investigations^14^ and atom probe tomography measurements^13^,^16^,^17^,^31^ proved the presence of Sr atoms within the eutectic Si as co-clusters with Al and Si (SrAl_2_Si_2_). The co-clustering has been explained to happen due to solute entrapment during the solidification process.

The region marked with the yellow dashed square in Fig. 1(a), has been subjected to a geometric phase analysis (GPA) which provided insight into the strain field distribution in the vicinity of interstitial strontium columns (strain maps contained in Supplementary Material Figure S.3). We noticed a lattice dilatation on the lateral sides within 2% and a compressive strain within 3% above and below strontium columns. The shear strain surrounding the Sr columns and along the twin growth direction does not exceed 0.5%. The strain field produced by the column induced reduced intensities at the twin end dislocations.

### A*b-Initio* calculations based on plane-wave density functional theory

**Figure 2:**
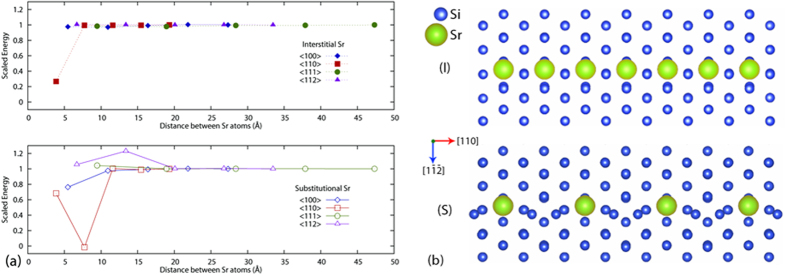
(**a**) Variation of the system energy with the distance between Sr atoms in the directions indicated in the legend. The vertical axis represents the excess energy above the pure Si lattice energy. This excess energy has been scaled by the long distance limit for each of the defects, which is 2.296 ± 0.072 eV for the interstitial case and 1.203 ± 0.100 eV for the substitutional case (with the error bounds resulting from differing k-point grids). Open and filled symbols correspond to substitutional and interstitial Sr, respectively. (**b**) Atomic configurations of the interstitial and substitutional <110> Sr columns, respectively, corresponding to the lowest energy states indicated by (I) and (S) in (**a**).

*Ab-initio* plane-wave density functional theory (DFT) modeling (confer to Methods and Supplementary Material) was performed to help answer the following questions: i) why does Sr appear to organize in <110> interstitial columns only, and ii) whether the preferential location of Sr at twin boundaries energetically favorable. Figure 2(a) shows the system energy variation with the distance between two Sr atoms in the same column for both substitutional (open symbols) and interstitial (filled symbols) cases when aligned in the <100>, <110>, <111> and <112> crystallographic directions. Figure 2(b) presents the atomic configuration for both interstitial and substitutional cases of lowest energy (strongest Sr-Sr bonding) marked by (I) and (S) in Fig. 2(a), viewed along the <111> direction. The energy reported is the excess relative to the pure Si model.

The following observations were made: 1) a binding energy between Sr atoms only exists when the column is oriented in the <110> direction of the Si lattice; 2) if Sr is substitutional, the strongest binding with Sr occurs in the second nearest neighbor position, i.e. with the respective column containing 50% Si and 50% Sr, Fig. 2(b) bottom; 3) if Sr is interstitial, the strongest binding occurs in the nearest neighbor position and hence interstitial Sr columns are composed entirely from Sr atoms, Fig. 2(b) top; 4) the interaction is short ranged, essentially vanishing beyond approximately 20 Å.

These observations are in agreement with our microscopy results, which show Sr columns oriented exclusively in the <110> direction and provide additional insight into the structure of these columns. The data are also in qualitative agreement with data from Yue *et al*.^26^, which show that Sr binds preferentially with Si. The real situation, however, is somewhere between fixed volume (zero dilatation) and zero pressure conditions. DFT calculations performed under zero pressure conditions provide results which differ from the values reported in Fig. 2(a) by less than 0.1 eV and all conclusions listed above remain valid.

**Figure 3:**
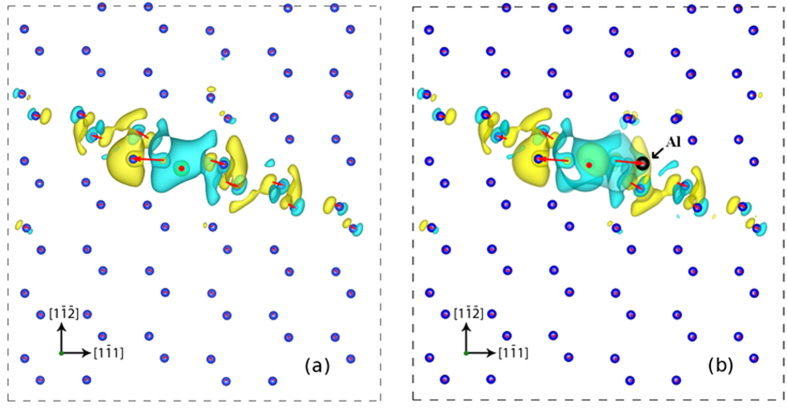
Charge density difference plot for the Si-Sr (**a**) and Si-Sr-Al (**b**) systems, with Si atoms (blue) and a <110> interstitial Sr column (marked by a red dot in the center of the image) in the {110} projection (yellow - regions of 
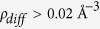
 and cyan - regions of 
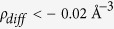
). The simulation cell size is 2×3 Si unit cells (18.9 Å × 20.1 Å) in the plane of the image. Superimposed on these images is the vector displacement field (shown by red arrows) of the Si atoms relative to the pure Si case (the tails of the arrows correspond to the location of the atoms in a perfect Si crystal). The charge perturbation is aligned with the <1–12> direction, while atomic displacements indicate incipient twinning. The position of the Al atom is shown in (**b**) with a black arrow. The displacement field and the charge density difference in (**b**) correspond to the {110} atomic plane containing the Al atom.

As discussed in refs 13,16,17,31, Al was reported to exist in Si in presence of Sr. It has been also suggested that the concentration of Al increases in proportion to that of Sr^17^. This indicates that some level of binding of Al and Sr in the Si crystal exists. To verify this possibility, we performed DFT simulations of the lowest energy interstitial Sr column (Fig. 2(b)) in the vicinity of which an Al atom was placed in several substitutional positions, in separate simulations. The model contains 5 Sr atoms and periodic boundary conditions are used in the direction of the Sr column. Therefore, the configuration corresponds to a ratio of 1:5 between the Al and Sr concentrations. The model is sufficiently large in the direction parallel to the Sr column to prevent the interaction of the Al atom with its images. The reference configuration for this situation is the model with the same Sr column and the Al atom located far from it. Several positions of the Al atom in the first ring of neighbors of the Sr column were considered. The configuration with the lowest energy is shown in Fig. 3(b). The Sr column is in the center of the image and marked with a red dot, while the position of the Al atom is indicated with the black arrow. The binding energy of the Al atom to the Sr column for this configuration is 0.26 eV. All other configurations investigated have energies smaller than the reference, but the binding energy is smaller than 0.26 eV. This indicates that substitutional Al does bind with the interstitial Sr column in Si and justifies the observations reported by Barrirero *et al*.^17^.

In order to investigate the interaction of the Sr column with the twin, we compare three systems: i) a perfect Si lattice with two successive first order twins in the {111} <112> system, ii) the above structure with two interstitial Sr columns oriented in the <110> direction located at the two twin boundaries, and iii) a perfect Si lattice without the twin but with the same Sr columns as in ii). In these models, the twin boundaries are of {111} type and their intersection with the <110> projection is the <112> direction, as shown in Fig. 1(a).

We denote the energies corresponding to cases i), ii) and iii) as E_i_, E_ii_ and E_iii_, respectively. The reference energy is that of a perfect crystal of Si with the same number of atoms, E_p_. The difference E_i_ − E_p_, which represents the energetic cost of placing a single twin boundary in the Si model, results in 0.0067 eV/Å^2^, which is equivalent to 0.224 eV/Å for the model of 33.47 Å width (length of the column). The cost of introducing a twin boundary and a Sr column located on it, E_ii_ − E_p_, yields only 0.114 eV/Å. The energy difference E_iii_ − E_p_, which represents the cost of placing a single Sr column in the pure Si model, comes out as 0.133 eV/Å per column. This means that it is energetically favorable for Sr to diffuse to the twin boundary, once a twin is already formed and Sr is located away from it. On the other hand, if an interstitial Sr column exists in un-twinned Si, the formation of a twin starting exactly from this column is energetically favorable too. In this case the energy decreases by E_ii_ − E_iii_ = −0.019 eV/Å. These conclusions do not change if the zero pressure correction is applied. Specifically, the corrected values for E_i_ − E_p_, E_ii_ − E_p_ and E_iii_ − E_p_ become 0.0067 eV/Å^2^, 0.089 eV/Å and 0.111 eV/Å, respectively, while the corrected value of E_ii_ − E_iii_ gives −0.022 eV/Å. These energy differences are reported in eV per Å of column length (in the <110> direction). Out of this result we conclude that a thermodynamic driving force for twin formation at Sr columns exists. However the analysis does not provide the barrier for nucleation, which has to be overcome by thermal activation. The barrier can be evaluated using, for example, the nudged elastic band method, in atomistic simulation. However, this is no feasible at this time since empirical potential do not exist for this system.

The charge density change caused by the addition of interstitial Sr, *ρ*_*diff*_,


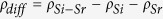


is plotted in the {110} projection in Fig. 3, where yellow indicates regions of 
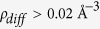
 while cyan indicates regions of 
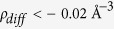
. For this calculation, a Si-Sr system containing a <110> column of interstitial Sr in un-twinned Si is considered (Fig. 3(a)). The combined Sr-Si electron density distribution 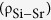 is then computed in VASP (see Methods) and the densities for the pure silicon system and that for an isolated Sr column are subtracted from it.

The position of the Sr column is indicated by the red dot. Figure 3(a) suggests that the electronic charge distortion extends along a <112> direction and the spatial extent of the perturbation (for the threshold used here) is well within 20 Å, which is the approximate size of the simulation cell. The possible cause lies in the interaction of the Sr atom with the first Si neighbors (the two smaller nearest left arrows on the figure), which induces an orbital hybridization in the <112> direction forcing the nearest Si atom to adopt an approximate planar sp^2^–like bonding scheme with a strong *p*_*z*_ character.

It is also of interest to discuss the displacement field in the vicinity of the Sr column. The red arrows in Fig. 3(a) show a vector plot indicating atomic displacements in the model. A twinning-like displacement is observed, with the nearest neighbor Si dumbbells rotating in the twinning direction. This is in agreement with the observation in Fig. 1(a), in which the nearest neighbor Si dumbbells are fully rotated and take the “star-like” configuration described above. These results provide support to the concept that the bright Sr column in Fig. 1 serves as nucleation center for the observed twins.

In this context, it is useful to inquire whether the presence of Al in the vicinity of the Sr column has any impact on the charge redistribution and displacement field. To address this question, we repeated the simulations using the model described above, in which an Al atom is placed next to the Sr column in the lowest energy configuration identified. Figure 3(b) shows the displacements and charge redistribution for the model with Al and in the plane perpendicular to the Sr column and containing the Al atom. Figures 3(a,b) indicate that the presence of Al (in the 1:5 ratio to Sr) leaves all conclusions discussed above unchanged. The displacements are essentially identical to those of the model without Al with the exception of one nearest neighbor of the Sr column which contain the Al atom. The charge redistribution is almost identical to that observed in the model without Al (Fig. 3(a)).

## Discussion

Among all modifying elements used in the industry, Sr and Na are the most effective in producing multiple twins. Since neither TPRE nor the IIT concept explain why for example Yb or other rare earth elements which have suitable radius ratio (for Yb this value is ~1.646), does not produce the expected twinning, other aspects have to be taken into consideration. It has been observed experimentally that when Yb atoms occupy substitutional positions in the crystal structure of eutectic Si replacing full columns (eg. bonding in the first neighbor) have no consequence on the twin formation^15^,^23^ which leads to the fibrous morphology, but forms intermetallic phases in the Al matrix.

Europium, on the other hand, was observed to modify the eutectic Si^18^. The reason for that seems to be the binding in the second nearest neighbor in the substitutional case (1/2 Eu occupancy) which leads to a planar sp^2^ arrangement. The segregation energy has been calculated to be higher, favoring the solute adsorption and entrapment during solidification which results in formation of Al_2_Si_2_Eu or Eu-rich clusters with a low number of atoms and multiple twinning mainly due to simultaneously poisoning of TPRE and IIT mechanism.

Although in the case of Sr the above mentioned formation of intermetallic phases and Sr-rich clusters might also happen^14^, the present study revealed an unexpected characteristic of the Sr atoms in Si crystal, namely their self-organization in interstitial columns. These columns are aligned in a defined orientation <110> and always at the edge of a twin and not only at the intersection of many twins as in case of Eu. The substitutional arrangement with bonding in the second neighbor seems to be similar to that of Eu. The energy calculation, however, indicates this as unlikely in Sr case since the energy difference is higher than in the interstitial case (Fig. 2) and the system should be stable for the minimum value. These observations are in full agreement with calculations reported in ref. 32 which shows that the lowest energy configuration of Sr on the Si (001) surface is characterized by interstitial rows of Sr formed in the <100> direction. Therefore the twinning mechanisms of Sr and Eu are fundamentally different. While in case of Eu twinning happens due to adsorption of atomic columns ahead of solidification front (poisoning of TPRE and IIT mechanism), in case of Sr twinning can also happen in the solid state. Yet, the diffusion rate doesn’t need to be very high, the Sr interstitial columns can also form slowly (heat treatment) assisted by thermal vacancies diffusion and the driving force for twin formation has been indicated by our *ab-initio* calculations. The occurrence of Al in the vicinity of Sr, even if a binding exists, does not change significantly the displacement field and the charge perturbation induced by the presence of the Sr columns. This supports the concept that twinning is mainly associated with the self-organization of Sr atoms in interstitial columns.

In conclusion, we have proven both experimentally and by *ab-initio* simulations that Sr atoms in Si tend to self-organize in columns aligned in the <110> direction. These columns have a binding energy with twin boundaries, which explains the preferential location of Sr at such boundaries. Importantly, if Sr columns form first, twins may nucleate from such sites. This implies that Sr may lead to twinning in the solid state, which is a new concept within the IIT framework which generally assumes that twins form during the growth of precipitates. This effect and the findings will be also useful for research of nano-dimensioned silicon based materials where formation of quantum dots and quantum walls in preferential directions is desired.

## Methods

### Sample preparation

An Al-Si alloy with 5 wt.% Si and 0.02 wt.% Sr was prepared by arc melting and subsequent melt spinning. For the Scanning Transmission Electron Microscopy (STEM) investigation, the samples were prepared by Ar milling at a constant temperature of −10 °C, using a cold stage. Further information is provided in the Supplementary Material.

### High resolution HAADF STEM imaging and EDX investigations

STEM images and analytical investigations were performed with a probe aberration-corrected microscope (FEI Titan3G 60–300 kV) equipped with a FEI Super-X (Chemi-STEM technology −0.7 sr collection angle)^33^ and a Dual Electron Energy Loss Spectroscopy (EELS) - Gatan Imaging Filter (GIF) Quantum^34^. For atomic resolution, the aberration-corrector element was optimized for an aberration-free zone of 25 mrad at 300 kV and of 23 mrad at 60 kV with a 50 μm condenser aperture. The beam convergence was 19.6 mrad for 300 kV and 19.9 mrad for 60 kV. High-angular annular dark-field (HAADF) and annular dark-field (ADF) detectors were used to acquire the high resolution STEM (HR STEM) micrographs. The cumulative EDX (energy-dispersive X-ray spectroscopy) spectrum image acquisition parameters were: 60 kV, 40 pA, 35 pixels × 35 pixels × 2048 channels with a pixel size of 23 pm, 0.1 sec pro spectrum. The thickness of the investigated particles was calculated after tilting using EELS (electron energy loss spectroscopy) point analysis. EELS analysis of Sr columns could not be involved since the major edges of Sr (L_3_ at 1940 eV and L_2_ at 2007 eV) lie in a region with very low signal to noise ratio. Trace elements detection at such high energy losses requires very long acquisition times and consequently higher dose, giving rise to a possible extraction of Sr atoms from the solid. On the other hand, the corresponding Sr M_45_ edge, featuring higher cross-sections, yields poorer signal-to-background ratio (“delayed edge“), despite the higher cross-sections.

Only very thin regions of the specimen (200–800 Å) have been used for high resolution imaging and analytical investigations. Further information is provided in the Supplementary Material.

### Image analysis

The geometric phase analysis (GPA) was performed following the description given by Hÿtch^35^ and by using the plugin for Digital Micrograph developed by C. Koch. This method uses the Fourier transform of an image containing periodical signals (eg. atomic columns) to reveal the symmetrical pattern of strong frequency components, namely the ***g*** vectors of the Bragg reflexions. When two reflexions corresponding to different ***g*** vectors are masked, amplitude and phase images (positional information with respect to a reference) can be generated; therefore the displacement and/or strain maps can be calculated. Care was taken in order to minimize the scanning artefacts: the stability of both the microscope and of the DigiScann device has been checked, and the experiment and acquisition time has been correspondingly chosen.

### Image simulation

QSTEM simulations were performed with the QSTEM code developed by C. Koch (2002)^36^. Pure Si-columns contain 193 lattice spacing corresponding to a sample thickness of 735 Å. For the simulation at 300 kV voltage with a beam convergence of 19.6 mrad, spherical aberration was set at 0.04 mm and defocus at −10.9 nm.

### Density functional theory calculations

Plane-wave density functional theory (DFT) calculations were performed using the Perdew-Burke-Ernzerhof (PBE) exchange-correlation functional^37^ and generalized gradient approximation (GGA) implemented in VASP^38^,^39^. Further information is provided in the Supplementary Material.

## Additional Information

**How to cite this article**: Albu, M. *et al*. Self-organized Sr leads to solid state twinning in nano-scaled eutectic Si phase. *Sci. Rep.* **6**, 31635; doi: 10.1038/srep31635 (2016).

## Supplementary Material

Supplementary Information
