# Supplementary material for: Self-organized Sr leads to solid state twinning in nano-scaled eutectic Si phase
Source: Sci Rep. 2016 Aug 16;6:31635. doi: 10.1038/srep31635 (PMC4985832; doi:10.1038/srep31635)
Supplement: Supplementary Information [file srep31635-s1.pdf]

**Supplementary Material:**

# Self-organized Sr leads to solid state twinning in nano-scaled eutectic Si phase

M. Albu[1*], A. Pal[3], C. Gspan[1], R.C. Picu[3], F. Hofer[1,2] and G. Kothleitner[1,2*]

1. Graz Centre of Electron Microscopy, Steyrergasse 17/III, 8010 Graz, Austria

2. Institute for Electron Microscopy and Nanoanalysis, Graz University of Technology, Steyrergasse 17/III, 8010 Graz, Austria

3. Department of Mechanical, Aerospace and Nuclear Engineering, Rensselaer Polytechnic Institute, Troy, NY 12180, USA

email: mihaela.albu@felmi-zfe.at, gerald.kothleitner@felmi-zfe.at

## Sample preparation

5N high purity Al (99.998 wt.% Al, produced by Hydro Aluminium High Purity GmbH, Grevenbroich, Germany), 5N high purity Si (Siltronic AG, Burghausen, Germany) and Al-4Sr master alloy (Al-3.59 wt.% Sr prealloy manufactured by using 4N Al + 99 wt.% Sr - Johnson Matthey Plc, London, U.K.) were used for this purpose. The experimental details concerning sample preparation, arc melting and melt-spinning are described in [1,2].

## STEM Imaging

The Titan microscope is equipped with X-FEG Schottky field-emission electron source, FEI Super-X detector (Chemi-STEM technology) consisting of four separate silicon drift detectors (0.7 sr collection angle) and Dual Electron Energy Loss Spectroscopy (EELS) - Gatan Imaging Filter (GIF) Quantum.

Eutectic Si particles of approximate 200 – 300 nm wide and 200 - 800 Å thick contained in the Al-Si alloy with 5 wt.% Si and 0.02 wt.% Sr, were tilted in the $\{111\}_{Si}\langle 110\rangle_{Si}//\{111\}_{?-Al}\langle 110\rangle_{?-Al}$ direction.

Imaging of strontium columns by STEM HAADF (300 kV) within the Si eutectic phase was strongly hampered by Sr diffusion which are likely to diffuse fast on the Si surface ($3*10^3$ Å/s on the Si (111) surface) due to thermal activation during irradiation with the beam[3]. Thus, the atoms situated at or close to either surface of the sample are likely to be extracted and should move on the surface,

resulting in a drastic reduction of the Sr-columns intensity in the HAADF high resolution STEM images. Therefore, only a very quick acquisition (~30 s at 300 kV) preserved their initial contrast in the HAADF image.

In order to preserve the number of Sr atoms in the region investigated by X-ray Spectrum Imaging, low dose conditions measurements were needed: a voltage of 60 kV with a low beam current of ~40 pA and cumulative measurements (seven subsequent spectrum images, 0.01 s pro spectrum, total acquisition time of 15s pro SI).

Electron energy loss spectrometry (EELS) could not be used in this investigation since Sr presents a major edge at very high energy losses: $L_3$ at 1940 eV and $L_2$ at 2007 eV. In this region the signal to noise ratio is very low making the detection of trace elements in low dose conditions (very short acquisition times) unreliable.

Experimental results

Figure S1 shows two further examples of EDX investigations of bright spots at the twin end representing typical findings from such areas. The spectra present the Al, Si, Cu, and Sr signals. Relative quantification indicates concentrations of Al - 14.9 at%, Si - 82.6 at% and Sr- 2.5 at%. We note that the amount of Al is the same in all regions over all investigated particles within an error of 0.5 at%, regardless of twins or interstitial Sr columns.

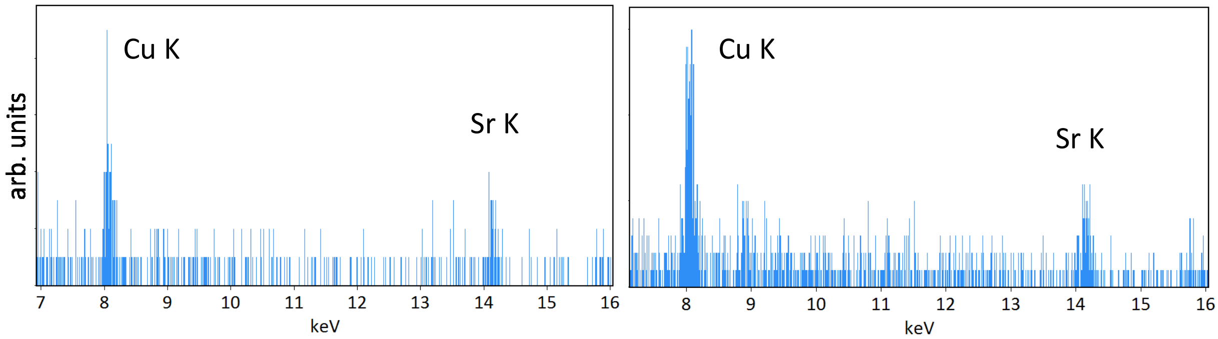


**Figure S 1**. Typical EDX spectra acquired from interstitial Sr columns (Spectrum images at 60 kV) as marked with yellow arrows in Fig. 1(a). Cu-signal comes from the TEM holder.

Image simulation

An examination of the HAADF intensities around the interstitial Sr columns reveals a clearly visible variation (not induced by beam damage) in the Si dumbbells intensity. To clarify the relative intensity at Si and Sr sites, we performed image simulations (QSTEM) of a fictitious Si lattice containing a Sr

column located either interstitially or substitutionally – Fig. S 2(a) and (b) respectively, and for Al located substitutionally (10, 77, 193 atoms) in a Si column nearby Sr -indicated with an yellow arrow on Fig. S 2(c). The sample thickness is 735 Å (193 Si lattice spaces in the column).

The general rule for identification of trace signals above image- or spectra- noise in an experiment is that the signal to noise ratio should be equal or higher than 3. However, the simulated images do not contain experimental noise; therefore the threshold contrast was calculated relative to Si columns. Given the intensity fluctuations in the experimental images (within 10 %) due to detector sensitivity parameter and local chemical perturbations, e.g. presence of possible vacancies or substitutional Al atoms in the Si columns, we considered values for the intensity ratio $I_{Sr}/I_{Si}$ higher than 1.1. This condition has been reached by 4 Sr atoms in interstitial position ($I_{Sr}/I_{Si}$ = 1.22) and 10 atoms in substitutional positions ($I_{Sr}/I_{Si}$ = 1.18). Therefore, we conclude that it is more difficult to identify Sr located substitutionally without image processing, unless the number of Sr atoms in the Si columns is higher than 10.

The contrast relative to the surrounding Si columns only decreases with 7 % if Si atoms are replaced by 10 Al atoms in the column (corresponds to the $SrAl_2$ – phase) while in case of 100 % (193 atoms) replacement the decrease is of 30 %. However we note a definitely lower contrast in two Si columns at the right site of the interstitial Sr in Fig. 1(a) top and bottom and the Si column at the middle right site of Sr disappears.

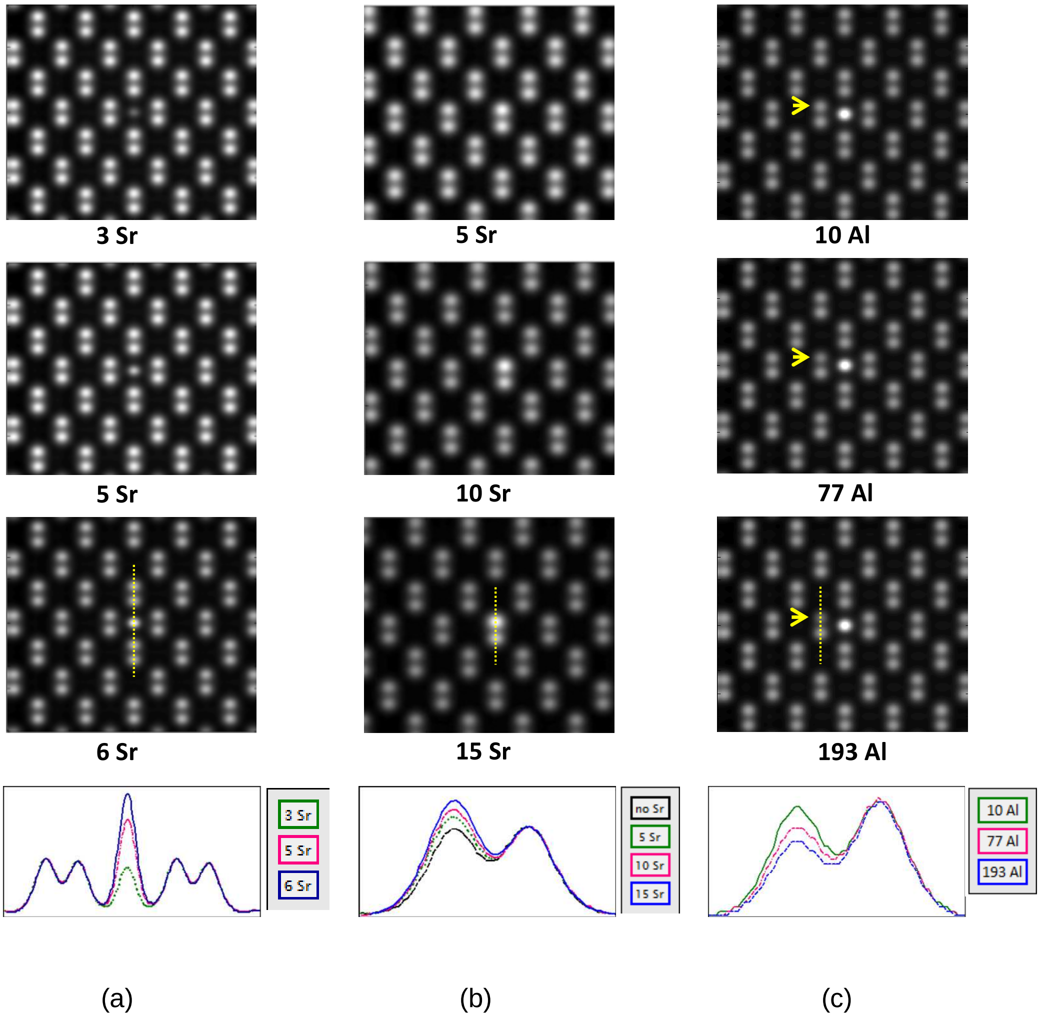


**Figure S 2**. **S**imulations for a qualitative interpretation of the HAADF image contrast with QSTEM. (a) Sr positioned interstitially, and (b) Sr positioned substitutionally in the Si crystal. The values represent the number of Sr atoms in each column (located in the center of the simulated domain). The various curves correspond to Sr columns containing different numbers of atoms. (c) Al atoms replacing Si atoms in a column next to Sr interstitial column: 5% (10 atoms), 40% (77 atoms) and 100% (193 atoms). Intensity profiles along the indicated dashed yellow lines are also shown.

<u>Geometric Phase Analysis</u>

This method uses the Fourier transform of an image containing periodical signals (eg. atomic columns) to reveal the symmetrical pattern of strong frequency components, namely the ***g*** vectors of the Bragg reflexions. When two reflexions corresponding to different ***g*** vectors are masked, amplitude and phase images (positional information with respect to a reference) can be generated; therefore the displacement and/or strain maps can be calculated.

Figure S 3 shows the HAADF image (a) along with the Fourier transform (b) and the respective strain tensors (c-f) calculated by using the GPA method.

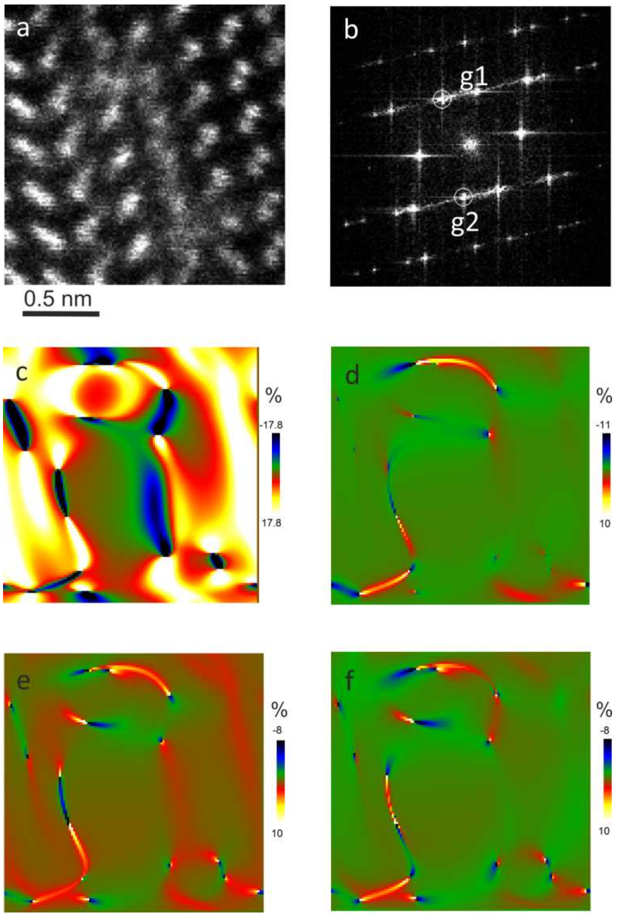


**Figure S 3.** (a) High resolution STEM – HAADF image of Si and Sr columns; (b) Fast Fourier Transform of the HAADF picture (zoom in); strain tensors in xx – direction (c), in yy – direction (d), in xy –shared direction (e) and, in yx – direction (f).

*Ab-initio* plane-wave density functional theory (DFT) calculations

As described in Methods, *ab-initio* plane-wave density functional theory (DFT) calculations have been performed by using the Perdew-Burke-Ernzerhof (PBE) exchange-correlation functional and generalized gradient approximation (GGA) implemented in VASP. An energy cut-off of 318 eV was used for the plane-wave basis set, and the ions were relaxed using projector augmented wave (PAW) pseudo-potentials to within a force-tolerance of 0.01 eV/Å [4,5]. Fine k-point mesh grids were used to sample the Brillouin zone. Images were developed using VESTA [6].

The interaction energy between Sr located either interstitially, at octahedral sites, or substitutionally was computed using fully periodic super-cells with the Si crystal oriented with the

<110>, <111> and <112> axes parallel to the simulation cell edges. Interaction energies along a specific crystallographic direction were computed by placing a single Sr atom in a large super-cell and varying the super-cell dimension (number of lattice unit-cells) in the respective direction. This allowed control over the line density of Sr atoms along a particular periodic direction, while keeping the model size and hence the Sr-Sr distances in the other two directions large enough to preclude interactions. Prior tests showed that if the distance between two Sr atoms is larger than 20 Å (in any crystal direction), the interaction vanishes or is too weak to be evaluated. The number of atoms in these models ranged from 160 to 180. The simulations are performed at fixed volume of the super-cell, corresponding to the volume of an equivalent model of pure Si at zero pressure and temperature. Considering the super-cell dimensions in the direction in which the Sr-Sr interaction is computed equal to the interatomic distance in the respective crystallographic direction, the model represents a column containing only Sr atoms. Otherwise, the column contains Si as well.

However, the real situation of the calculations presented in Figure 2 is somewhere between fixed volume (zero dilatation) and zero pressure conditions. Performing DFT simulations while relaxing the super-cell in order to achieve zero pressure conditions, although feasible, it is much more computationally demanding. Therefore, we estimate the binding energies at zero pressure by subtracting from each computed energy the work associated with volumetric relaxation, i.e. $p^2V/2B$, where p is the pressure in the super-cell computed under constant volume conditions, V is the volume of the super-cell and B is the bulk modulus of Si (taken here equal to 89 GPa). The results differ from the values reported in Figure 2 (a) by less than 0.1 eV and all conclusions listed above remain valid.

The energy calculation data are in qualitative agreement with the results given by Yue (2013) which indicate that Sr binds preferentially with Si. However, some degree of screening should occur in the disordered, melt phase and in presence of Al since the pair distribution function of Sr does not show a peak at distances corresponding to the first or second neighbor positions of the crystal. This difference can also be due to the lack of statistics in the liquid containing very few Sr atoms.

For the interaction of the Sr column with the twin we considered the twins to be two lattice spacing thick as observed experimentally in Figure 1. An important requirement, considering that they are the thinnest possible twins, is that the energetic interaction between twin boundaries is zero. The interstitial Sr columns are placed at the two twin boundaries and are composed from Sr atoms located in the nearest neighbor position, which corresponds to the interstitial Sr column configuration discussed above (Figure 2 (a)). The distance between columns in the {110} plane is sufficiently large to prohibit significant direct interaction between them. Periodic boundary

conditions are used in all directions and the model size is large enough to preclude significant interactions of Sr columns and their images or of twin boundaries.


References:

1. Zarif, M., McKay, B. & Schumacher, P., Metall Mater. Trans. A **42**, 1684–1691, (2011).

2. Li, J. et al, Acta. Mater. **72**, 80–89 (2014).

3. Zhachuk, R., Teys, S., Olshanetsky, B. & Pereira, S., Appl. Phys. Lett. **95**, 061901 (2009).

4. Blöchl, P. E., Phys. Rev. B **50**, 17953 (1994).

5. Kresse, G. & Joubert, D., Phys. Rev. B **59**, 1758 (1999).

6. Momma, K. & Izumi, F., J. Appl. Crystallogr. **44**, 1272-1276 (2011).
